# Systematic Examination of Infant Size and Growth Metrics as Risk Factors for Overweight in Young Adulthood

**DOI:** 10.1371/journal.pone.0066994

**Published:** 2013-06-20

**Authors:** Andrew O. Odegaard, Audrey C. Choh, Ramzi W. Nahhas, Bradford Towne, Stefan A. Czerwinski, Ellen W. Demerath

**Affiliations:** 1 Division of Epidemiology and Community Health, School of Public Health, University of Minnesota, Minneapolis, Minnesota, United States of America; 2 Lifespan Health Research Center, Boonshoft School of Medicine, Wright State University, Dayton, Ohio, United States of America; Universidade Federal do Acre (Federal University of Acre), Brazil

## Abstract

**Objective:**

To systematically examine infant size and growth, according to the 2006 WHO infant growth standards, as risk factors for overweight status in young adulthood in a historical cohort. Specifically, to assess: Whether accounting for length (weight-for-length) provides a different picture of risk than weight-for-age, intervals of rapid growth in both weight-for-age and weight-for-length metrics, and what particular target ages for infant size and intervals of rapid growth associate most strongly with overweight as a young adult.

**Patients/Methods:**

Data analysis of 422 appropriate for gestational age white singleton infants enrolled in the Fels Longitudinal Study. Odds ratios (OR) for overweight and obesity in young adulthood (age 20–29) were calculated using logistic regression models for the metrics at each target age (0, 1, 3, 6, 9, 12, 18, 24 months) comparing ≥85^th^ v. <85^th^ percentile, as well as rapid growth (Δ≥0.67 Z-score) through target age intervals. Models accounted for both maternal and paternal BMI.

**Results:**

Infants ≥85^th^ percentile of weight-for-age at each target age (except 3 months) had a greater odds of being overweight as a young adult. After accounting for length (weight-for-length) this association was limited to 12, and 18 months. Rapid weight-for-age growth was infrequently associated with overweight as a young adult. Rapid weight-for-length growth from 0 to 24 months, 1 to 6, 9, 12, 18, and 24 months and from 3 to 9, 12, 18, and 24 months was strongly associated with overweight status as a young adult.

**Conclusions:**

The WHO weight-for-length metric associates differently with risk of being overweight as a young adult compared to weight-for-age. Intervals of rapid weight-for-length growth ranging from months (0–24), (1–12, 18, and 24) and (3–9, and 12) displayed the largest OR for being overweight as a young adult.

## Introduction

Extensive research has demonstrated overweight and obese adults experience a greater risk for a spectrum of health outcomes.[1] A problematic aspect of carrying excess weight in adulthood is the difficulty in transitioning to a maintained healthier weight range from overweight or obese statuses.[2] Life-course studies have identified infancy as a potential critical period that affects risk for excess weight and common related chronic diseases as an adult; thus public health approaches for prevention have targeted this upstream age range.[3]

A number of studies have examined size and growth during different periods of infancy with later obesity, and systematic reviews have concluded larger infant size and rapid growth are associated with later obesity in childhood and adulthood.[4], [5], [6] Despite the consistency of these findings, the majority of the studies have used child or adolescent obesity as the outcome rather than adulthood, used internal or descriptive growth charts as a reference rather than the currently recommended 2006 World Health Organization growth standards, reported data at limited time points during infancy (e.g. birth, 4, 12, or 24 months), and did not have both maternal and paternal BMI for comparison and adjustment.[4], [5], [6], [7] Furthermore, the preponderance of studies used weight-for-age as a metric,[8] which provides information on energy balance, but does not completely inform on the nature of infant growth.

To begin to address these knowledge gaps, we aimed to systematically examine infant size and growth as risk factors for overweight status in young adulthood in a historical cohort according to the 2006 WHO infant growth standards. Specifically, we assessed: 1) Whether accounting for length (weight-for-length) at target ages provides a different picture of risk than weight-for-age, 2) intervals of rapid growth in both weight-for-age and weight-for-length metrics, 3) if particular target ages for infant size or intervals of rapid growth associate more strongly with overweight as a young adult, and 4) if parental BMI modifies the relationship between infant growth and overweight status as an adult.

## Methods

All protocols and informed consent documents were approved by the Wright State University and University of Minnesota Institutional Review Board. Parents provided written consent for their offspring's participation until seven years of age, at which point the children provided their additional assent. The study sample consisted of 422 appropriate for gestational age white singleton infants (215 males, 207 females) enrolled in the Fels Longitudinal Study. The Study began in 1929 as a study of individual variation in the growth and development of children,[9] and has continued as a study of early life antecedents of cardio-metabolic risk in adulthood.

For inclusion in this analysis, participants were required to have at least 3 serial weight and length measurements between the ages of 1 and 42 months, and a measured birth weight. Weight and recumbent length were collected at target ages of 0, 1, 3, 6, 12, 18, 24, 30, 36, and 42 months. For measurements over 24 months of age, standing height was used *in lieu* of recumbent length. Other inclusion criteria included a height and weight measurement between the ages of 20 and 29 years, as well as maternal and paternal age and BMI.

Missing growth data (<5% of subjects at any given time point) occurred randomly at the different target ages. To account for this we modeled the serial weight and length data for all children in a mixed effects regression model using a fourth-degree polynomial function of age, implemented in SAS PROC MIXED.[10] All age terms were included as both fixed and random effects, and models also included sex, birth year, birth year^2^, age-by-sex and age-by-birth year interactions as fixed effects. Age^3^ and age^4^ random effects were not significant for length and therefore dropped out of the models for length. Residual and influence diagnostics indicated that model assumptions were not violated and estimates of fixed effects were not overly influenced by any single individual. The correlations between observed and predicted values for all ages were high (r>0.98). A mixed-effects regression model results in a reduction in error compared to the use of individual regression models.[10]

From the mixed effects models, predicted weights and lengths at each specific target age from birth to 42 months were obtained ensuring a consistent sample size for weight and height at each target age and a reduction in error compared to the use of individual regression models.[10] These predicted values were then used to calculate weight-for-age, length-for-age, and weight-for-length Z-scores and their corresponding percentiles for target ages from birth- 24 months based on the World Health Organization growth standards.[11] The United States Centers for Disease Control (CDC) has recently recommended the adoption of the 2006 WHO growth charts as the standard for monitoring growth in United States children less than 24 months of age.[7] The WHO charts are based on data from infants exclusively breast fed for approximately the first 6 months of life, and are said to represent how healthy children should grow under optimal environmental and health conditions.[7] The prior recommended CDC growth charts are a growth reference that describe how certain children grew in a particular place and time.[7] Details on the development, interpretation, and inclusion criteria for the WHO growth standard population are found in Grummer-Strawn et al.[7]

Odds ratios (OR) for overweight and obesity in young adulthood were estimated using logistic regression models. We compared infants at or above the 85^th^ percentile (Z-score ≥1.036) for each metric at each target age to infants below the 85^th^ percentile. There was a threshold of no association with overweight or obesity in those below the 75^th^ or 85^th^ percentile upon detailed examination of the distribution (e.g. <50^th^, 50^th^–74^th^, 75^th^–84^th^, 85^th^–94^th^, 95^th^+). Covariates included sex, birth year, age at adult BMI assessment, gestational age at birth (calculated from maternal report of last menstrual period at the time of birth), and maternal age. Maternal and paternal BMI and paternal age were collected using the non-pregnancy weight closest to the birth date. We adjusted models examining weight-for-age or weight-for-length at subsequent ages for the WHO Z-score at birth in order to estimate associations at these ages that are distinct from associations due to size at birth. Further adjustment for parental BMI and ages occurred as well to demonstrate the influence these covariates had on the point estimates.

In addition, we categorized infants as having rapid, slow or gradual/normal growth according to Ong et al[12] definition of a clinically significant increment between different infant target ages where infants with an increase of 0.67 standard deviation score (SDS) or higher between various ages were defined as having rapid growth, those with decreases in SDS<−0.67 SDS were defined as having slow growth, and those with increments between −0.67 and +0.67 SDS were defined as having gradual growth over the interval. A±0.67 SDS change corresponds to a change in 1 major centile band (e.g 25^th^–50^th^, 50^th^–75^th^, etc) on a growth chart. In logistic regression models, with a similar pattern of adjustment as the status measures, we compared infants who experienced rapid growth to those who experienced slow or gradual growth since there was no evidence of any association of slow growth with excess weight. Sensitivity analyses stratifying by sex for both size and growth status were performed, as well as examination of secular trends by birth year. All analyses were carried out using SAS version 9.2.

## Results

**Figure 1:**
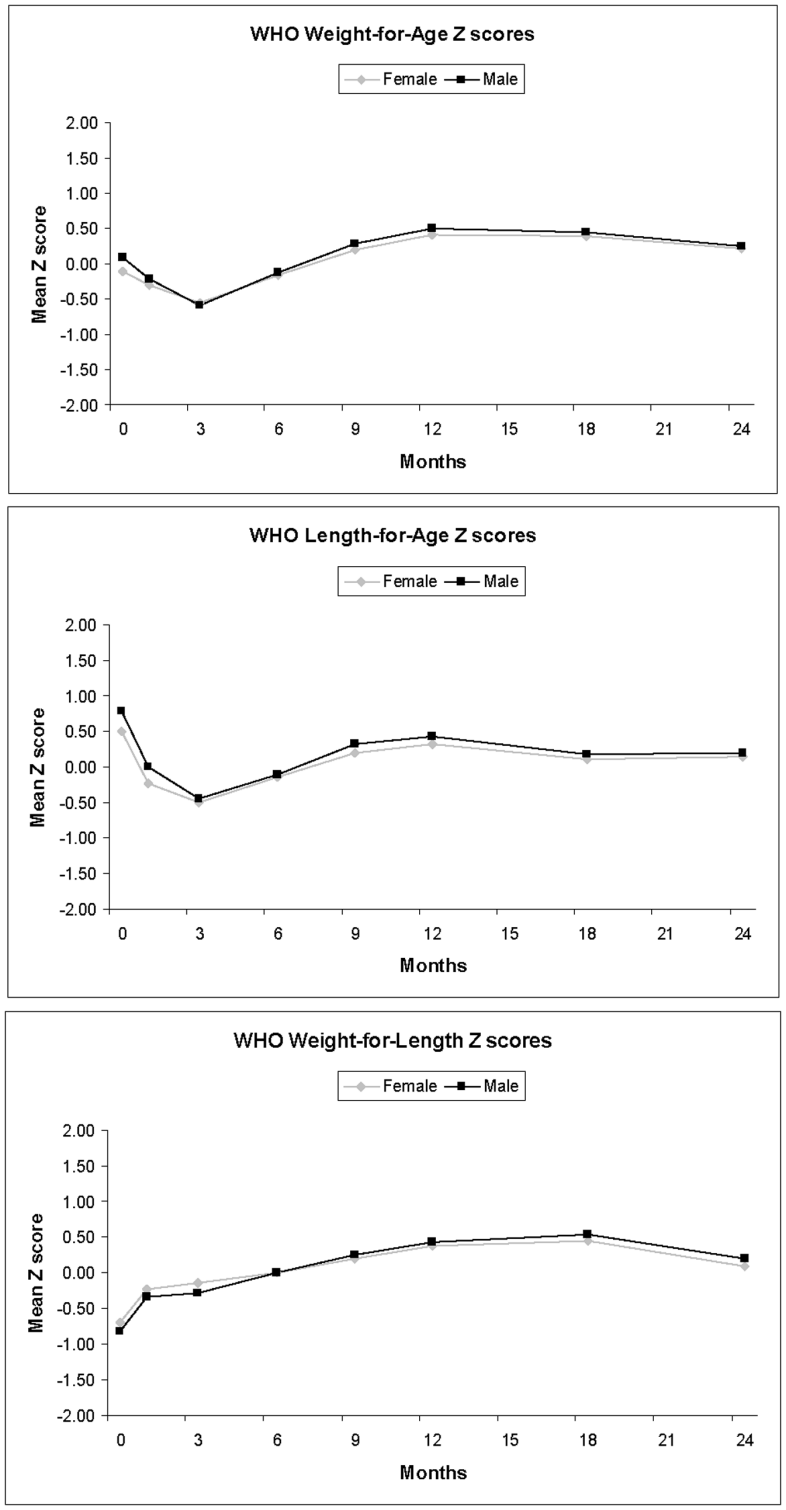
Mean WHO Infant Weight-for-Age, Length-for-Age, and Weight-For-Length Z scores by sex in the Fels Longitudinal study.

Of the 422 infants in the analysis 22 were obese (BMI ≥30 kg/m^2^) and 101 were overweight (BMI ≥25 kg/m^2^) as young adults (age 20–29). **Table 1** displays descriptive characteristics by sex of the study sample. **Figure 1** presents the mean WHO Z-scores for weight, length and weight-for-length by age. At birth, the mean Z-scores for weight are around 0 (the 50^th^ percentile) and then decline until 3 months, after which they begin to increase and level off slightly above the 50^th^ percentile at 12 months. At birth, the mean Z-scores for length are greater than the 50^th^ percentile and decline until 3 months and then experience a similar trend of increase to weight-for-age Z-scores thereafter. Due to greater birth lengths, the mean weight-for-length Z-scores were below the 50^th^ percentile at birth and then increased until 18 months.

**Table 1:** Population characteristics by sex.

	Male	Female
N	215	207
Adult BMI (kg/m^2^)	24.2 (1.7)	24.1 (2.1)
N overweight	60	41
N obese	11	11
Birth weight (kg)	3.47 (0.54)	3.26 (0.49)
Birth length (cm)	52.0 (2.1)	51.0 (1.9)
Gestational age (wk)	39.6 (1.9)	39.8 (1.9)
Mean birth year	1955 (14.4)	1955 (14.3)
aAge BMI adult (years)	24.2 (1.8)	24.0 (2.1)
bMaternal BMI (kg/m^2^)	22.8 (2.9)	22.4 (3.9)
Maternal age (years)	28.6 (5.4)	27.9 (5.4)
cPaternal BMI (kg/m^2^)	25.1 (3.9)	25.4 (4.2)
Paternal age (years)	35.8 (10.6)	35.0 (10.4)

All values Mean (SD).

a Age BMI adult = Age of adult anthropometric assessment.

Overweight =  BMI≥25 kg/m^2^.

Obese  =  BMI≥30 kg/m^2^.

b Maternal BMI =  Non-pregnant BMI nearest infant birth.

c Paternal BMI =  BMI nearest infant birth.

Maternal and Paternal age  =  Age at infant birth.

**Table 2** presents odds ratios (OR) and 95% confidence intervals (CI) from the logistic regression models examining high infancy WHO weight-for-age and weight-for-length (defined as ≥85^th^ percentile) with overweight in young adulthood. Across the spectrum of infancy, except for 3 months, a high weight-for-age measure is associated with a 2–3 fold increased odds of being overweight as a young adult. Upon accounting for length (weight-for-length), an association is only observed in later target ages, specifically at 12 and 18 months and suggestively at 9 and 24 months. The strongest association for weight-for-length was at 18 months, OR = 2.65, 95% CI (1.51–4.65).

**Table 2:** Odds ratio and 95% confidence interval of being overweight (BMI≥25 kg/m^2^) as a young-adult (age 20–29 years) according to percentile of the WHO infant growth standards: Infants ≥85^th^ percentile vs. <85^th^ percentile.

Metric: WHO Weight for Age					
**Outcome**	**Target Age (Months)**	**^a^N^ovwt^/ N^85th^**	**^1^OR (95% CI)**	**^2^OR (95% CI)**	**^3^OR (95% CI)**
**Overweight**	**0**	20/47	2.87 (1.48–5.58)	NA	2.02 (0.99–4.13)
	**1**	15/27	4.91 (2.13–11.36)	4.14 (1.60–10.69)	3.50 (1.28–9.53)
	**3**	3/9	NA	NA	NA
	**6**	14/34	2.45 (1.17–5.13)	2.03 (0.94–4.37)	2.40 (1.06–5.42)
	**9**	29/72	2.56 (1.48–4.43)	2.22 (1.22–4.01)	2.34 (1.24–4.43)
	**12**	37/98	2.39 (1.45–3.94)	2.12 (1.25–3.59)	2.17 (1.24–3.81)
	**18**	39/100	2.72 (1.65–4.47)	2.46 (1.47–4.12)	2.67 (1.54–4.64)
	**24**	30/70	3.04 (1.75–5.27)	2.71 (1.53–4.80)	2.91 (1.57–5.40)
**Metric: WHO Weight for length**					
	**0**	6/16	2.55 (0.87–7.44)	NA	2.13 (0.68–6.68)
	**1**	7/21	1.94 (0.74–5.09)	1.42 (0.45–4.45)	1.58 (0.47–5.30)
	**3**	4/15	1.29 (0.39–4.22)	0.92 (0.24–3.62)	0.99 (0.24–4.07)
	**6**	9/30	1.41 (0.62–3.22)	1.23 (0.52–2.91)	1.53 (0.62–3.78)
	**9**	17/51	1.79 (0.94–3.40)	1.72 (0.89–3.32)	1.92 (0.95–3.85)
	**12**	29/85	2.01 (1.18–3.42)	2.00 (1.16–3.45)	1.98 (1.11–3.52)
	**18**	38/102	2.68 (1.51–4.48)	2.70 (1.59–4.59)	2.65 (1.51–4.65)
	**24**	20/56	2.24 (1.19–4.23)	2.23 (1.15–4.32)	1.90 (0.93–3.87)

-Overall N = 422, 101 overweight young adults (BMI≥25 kg/m^2^).

- OR (95% CI) represents estimate for ≥85^th^ percentile by target age compared to <85^th^ percentile by target age (OR = 1.00).

- ^a^N^ovwt^/ N^85th^ =  Number who became overweight as young adults in the group who were ≥85^th^% at the target age.

- Model 1: *^1^OR (95% CI)*  =  Adjusted for sex, gestational age at birth, age at adulthood obesity assessment, birth year,

- Model 2: *^2^OR (95% CI)*  =  Model 1 further adjusted for birth WHO weight-for-age or weight-for-length Z-score.

- Model 3: ^3^ *OR (95% CI)*  =  Model 2 plus maternal and paternal age and BMI.

The associations were all independent of birth Z-scores and parental data. Analyses examining only those who were overweight in young adulthood (BMI 25–29.9) as the outcome, and thus excluding obese subjects showed subtly attenuated, and consistent results (data not presented). There was no evidence upon stratification by sex that the associations between infant weight-for-age, or weight-for-length and young adulthood overweight differed in girls and boys (data not presented). Length-for-age percentile was not associated with overweight or obesity at any infant target age (data not presented).

We also accounted for the historical nature of the cohort in stratified analyses of infants born before 1960, and 1960 and thereafter. We observed associations highly similar to the main results. However, associations tended to be stronger for those born 1960 and thereafter. For example, 18 month old infants with a high weight-for-age percentile (≥85^th^%) born pre-1960 observed a strong association with overweight (OR = 2.46, 95% CI 1.14–5.23), as did those born 1960 and after (OR = 3.76, 95% CI 1.52–9.33). The 1960 birth year was chosen to align with the approximate onset of the current obesity epidemic. We were unable to carry out stratified analyses for obesity as the outcome due to the small number of obese young adults producing unstable estimates.

**Table S1 in File S1** presents similar data to Table 2, but with obesity (BMI≥30 kg/m^2^) as the outcome. Infants with elevated weight-for-age at birth and 1 month display strong associations with obesity, and there is a suggestive association for the 24 month point. Elevated weight-for-length at 12, 18, and 24 months are the only target ages strongly associated with greater odds of obesity in young adulthood. Due to small numbers there were no data for earlier target ages. Similarly, the strongest association for weight-for-length was at the 18 month point.

**Table 3** presents the ORs and 95% CIs for being overweight in young adulthood according to rapid weight-for-length growth relative to infants with non-rapid growth. Rapid growth from birth to 24 months was associated with greatly increased odds of being overweight as a young adult. Rapid growth from 1 month to 6, 9, 12, 18, and 24 months was strongly associated with young adulthood overweight, with the strongest association at 1-12 months We also observed strong associations for rapid growth between the periods of 3 to 9, 12, 18, and 24 months. Further associations were observed between 6 to 12 and 18 months, but were not as strong as the other periods. **Table 4** presents similar data to table 3, but with weight-for-age rapid growth as the metric. The majority of intervals were not statistically significant, and the intervals that were, are more modest associations relative to weight-for-length rapid growth. The one exception was the range of 6–9 months, where 16 of 34 participants who increased a centile in weight-for-age became overweight, (OR = 4.71, 95% CI (1.86–11.94).

**Table 3:** Odds ratio and 95% confidence interval of being overweight (BMI≥25 kg/m^2^) as a young-adult (age 20–29 years) according to infant growth status between target ages: Rapid Weight-for-Length growth (Δ≥0.67 Z score) vs. Non-rapid Weight-for-Length growth (Δ<0.67 Z score).

Rapid Growth - Metric: WHO Weight for length			
**Outcome**	**Target Age (Months)**	**^a^N^ovwt^/N^rapid^**	***Rapid Growth*** **OR (95% CI)**
**Overweight**	**0–1**	20/119	0.68 (0.34–1.35)
	**0–3**	36/166	1.13 (0.61–2.09)
	**0–6**	53/222	1.67 (0.89–3.13)
	**0–9**	60/247	1.83 (0.96–3.49)
	**0–12**	66/272	1.93 (1.00–3.72)
	**0–18**	68/287	1.73 (0.90–3.29)
	**0–24**	67/239	4.12 (2.15–7.90)
	**1–3**	8/37	1.20 (0.45–3.19)
	**1–6**	31/111	2.38 (1.22–4.66)
	**1–9**	51/174	2.68 (1.44–5.01)
	**1–12**	62/202	4.40 (2.33–8.31)
	**1–18**	65/224	3.86 (2.07–7.19)
	**1–24**	51/163	3.24 (1.78–5.90)
	**3–6**	12/39	2.05 (0.84–5.00)
	**3–9**	44/132	3.70 (1.98–6.93)
	**3–12**	60/195	3.49 (1.94–6.28)
	**3–18**	64/213	2.67 (1.54–4.61)
	**3–24**	44/139	1.91 (1.11–3.31)
	**6–9**	3/6	NA
	**6–12**	25/78	1.97 (1.03–3.76)
	**6–18**	44/137	1.81 (1.06–3.08)
	**6–24**	26/80	1.39 (0.75–2.57)
	**12–24**	5/18	0.94 (0.28–3.18)

-Overall N = 422, 101 overweight young adults (BMI≥25 kg/m^2^).

-Target age represents the beginning and end point of the growth period, e.g. 0–1 is birth to1 month.

-Rapid infant growth: (Δ≥0.67 Z score of WHO weight-for-length standard) ∼ change in centile on growth chart.

- OR (95% CI) represents estimate for rapid growth (Δ≥0.67) between target ages compared to non-rapid growth (Δ<0.67 Z score) (OR = 1.00).

- ^a^N^ovwt^/ N^rapid^  =  Number who became overweight as young adults in the group who experienced rapid growth between target age points.

-All models adjusted for sex, gestational age at birth, age at adulthood obesity assessment, birth year, maternal and paternal age and BMI and birth weight-for-length Z-score.

**Table 4:** Odds ratio and 95% confidence interval of being overweight (BMI≥25 kg/m^2^) as a young-adult (age 20–29 years) according to infant growth status between target ages: Rapid Weight-for-Age growth (Δ≥0.67 Z score) vs. Non-rapid Weight-for-Age growth (Δ<0.67 Z score).

Rapid Growth - Metric: WHO Weight for Age			
Outcome	Target Age (Months)	^a^N^ovwt^/N^rapid^	*Rapid Growth*OR (95% CI)
**Overweight**	**0–1**	1/2	NA
	**0–3**	3/13	1.07 (0.27–4.25)
	**0–6**	19/73	1.69 (0.84–3.41)
	**0–9**	28/134	0.91 (0.50–1.68)
	**0–12**	42/170	1.60 (0.90–2.84)
	**0–18**	42/168	1.54 (0.88–2.71)
	**0–24**	37/138	2.04 (1.11–3.74)
	**1–3**	0/6	NA
	**1–6**	20/71	1.88 (0.93–3.77)
	**1–9**	40/165	1.46 (0.82–2.58)
	**1–12**	55/211	1.97 (1.12–3.46)
	**1–18**	54/208	1.55 (0.89–2.68)
	**1–24**	44/163	1.80 (1.02–3.18)
	**3–6**	24/76	2.20 (1.12–4.33)
	**3–9**	65/260	1.59 (0.91–2.79)
	**3–12**	76/305	1.65 (0.91–3.00)
	**3–18**	75/282	2.01 (1.12–3.60)
	**3–24**	61/222	1.77 (1.04–3.04)
	**6–9**	16/34	4.71 (1.86–11.94)
	**6–12**	44/151	1.78 (1.03–3.11)
	**6–18**	47/151	2.15 (1.26–3.69)
	**6–24**	35/117	1.59 (0.91–2.78)
	**12–24**	4/9	2.45 (0.53–11.27)

-Overall N = 422, 101 overweight young adults (BMI≥25 kg/m^2^).

-Target age represents the beginning and end point of the growth period, e.g. 0–1 is birth to1 month.

-Rapid infant growth: (Δ≥0.67 Z score of WHO weight-for-age standard) ∼ change in centile on growth chart.

- OR (95% CI) represents estimate for rapid growth (Δ≥0.67) between target ages compared to non-rapid growth (Δ<0.67 Z score) (OR = 1.00).

- ^a^N^ovwt^/ N^rapid^  =  Number who became overweight as young adults in the group who experienced rapid growth between target age points.

-All models adjusted for sex, gestational age at birth, age at adulthood obesity assessment, birth year, maternal and paternal age and BMI and birth weight-for-age Z-score.

When considering rapid weight-for-length growth with obesity as the outcome, similar trends were observed for the periods beginning at 1 and 3 months (**Table S2 in File S1**). However there was no association from any periods 6 months or on, and birth to 12 and 18 months also displayed significant associations. However, because of smaller number of obese young adults, and less precision the estimates require cautious interpretation. There was no association with obesity when categorizing rapid growth using WHO weight-for-age percentiles (**Table S3 in File S1**) or length-for-age percentiles (data not presented).

Lastly, in models that examined the association of maternal and paternal BMI with odds of offspring overweight as a young adult, strong positive associations were observed. Compared to infants with normal weight mothers, infants with overweight and obese mothers observed significantly increased odds of being overweight during young adulthood, (OR = 2.27, 95% CI 1.19–4.38), and obese (OR = 6.58, 95% CI 2.41–17.94). Similar positive, albeit not as strong associations were observed for infants with overweight and obese fathers relative to normal weight (OR = 2.35, 95% CI 1.37–4.00) for overweight and (OR = 3.69, 95% CI 1.55–8.79) for obesity. Results represent fully adjusted models including mutually accounting for paternal/maternal BMI and age.

## Discussion

In this systematic examination of infant size and growth, infants at target ages from birth to 2 years (except 3 months) at and above the 85^th^ percentile of the 2006 WHO infant growth standards of weight-for-age had a greater odds of being overweight as a young adult. After accounting for length (weight-for-length), significant associations were observed only at 12 and 18 months, and suggestively at 9 and 24 months. Rapid weight-for-length growth from birth to 24 months, and 1 month to 6 months and thereafter in infancy was strongly associated with carrying excess weight as a young adult. Similar strong associations were observed from 3 to 9 months and beyond. Rapid weight-for-age growth displayed more modest and inconsistent associations; and was not uniform between overweight and obesity outcomes in young adulthood. Thus, the data suggest the more complete metric in respect to infant size and growth, the WHO weight-for-length standard, may provide a different view of risk relative to weight-for age.

Overall, rapid growth intervals displayed stronger associations than static target-age measures and were independent of birth size. The strongest (based upon point estimate and confidence intervals) target-age association with young adulthood overweight status for weight-for-length was at the 18 month point, and for rapid growth was from the period 1–12 months. We also observed strong associations between parental BMI, including paternal, and odds of overweight status as an adult. These estimates were as strong as or stronger than the association of the infant growth metrics, yet the infant growth metrics were independent of parental BMI and the estimates were only subtly attenuated by adjustment.

In addition, the study results suggest the WHO 2006 infant growth standards have clinical utility since they strongly associate with a long term health outcome of public health significance. On the whole, this study provides a unique view of infant size, growth, and rapid growth relative to the literature base on the topic. Not only do our data suggest a wide swath of target ages and growth periods during infancy provide insight on risk of excess weight as a young adult, we also present results demonstrating the importance of accounting for length in studies of infant growth. Indeed, elevated weight-for-length percentile at birth did not predict overweight or obesity suggesting that greater birth weights need to be put in context of the length of the newborn when applying the 2006 WHO infant growth standards. Furthermore, when applying the 2006 WHO infant growth standards, our data suggest it may be prudent to consider rapid growth patterns beginning in the months (1 or 3) after birth when the infant has seemingly assorted on a certain growth trajectory.

Secondary to the logistic regression models, the mean WHO Z-scores for the separate metrics suggest growth faltering occurs when examining weight and length Z-scores from birth to 3 months of age before recovering and stabilizing. This was most pronounced in length Z-scores, which aligns with international surveys of infant growth utilizing the WHO standards showing faltering in length Z-scores up to 24 months before stabilizing.[13] Wright et al observed UK children with relatively high mean z-scores for birth weight and length compared with the WHO standards with rapid declines toward the WHO median by 2 weeks.[14] A higher proportion of U.S. infants displayed apparent “growth faltering” (i.e., decline of >2 Z-scores) in both weight and length from 0–6 months, followed by more rapid growth than the standards from 6–12 months of age.[15] Thus this individual trend of faltering at some time point relative to the WHO standards is not novel. This faltering may be explained by maternal stature at birth,[16] or due to feeding practices, as slower growth during the first 3 months may be due to formula feeding.[7] However, we are only able to speculate. The weight-for-length Z-scores do not exhibit this trend. These trends in the data may explain why we did not find any association with rapid or slow growth between individual length WHO Z-scores, moderate and inconsistent associations with weight-for-age rapid growth, but observed strongly significant findings with overall rapid growth utilizing weight-for-length Z-scores.

The systematic reviews examining size and growth during infancy are in consensus that there is an association between larger size and rapid growth during infancy with later obesity.[4], [5], [6] However, these reviews and conclusions better apply to obesity in childhood and adolescence than adulthood since the frequency of studies examining size or growth at some point in infancy with obesity in adulthood are scarce. To our knowledge this study is the only one to date to systematically examine the full range of infant growth, and growth patterns, thus providing further insight on this topic relative to the published literature. A handful of other studies have also addressed this topic with different approaches since the original reviews.

A Finnish study examining peak height and weight growth velocity from birth to two years, found that both were associated with adiposity at age 31.[17] A study from the Netherlands including a range of healthy participants of various birth sizes found that rapid infant growth (>0.67 SDS) in the first 3 months of life was significantly associated with increased adiposity and measures of cardio-metabolic risk at 18–24 years, suggesting that rapid growth may be pernicious regardless of birth weight.[18] McCarthy et al.[19] reported that birth weight did not predict adiposity in adulthood based on weight gain in childhood, but that increased weight gain velocity from 21 months through 5 years was associated with increased adiposity. In a study of formula-fed infants from Iowa, USA, weight gain during first week of life was associated with overweight status as a young adult.[20] A study of men and women in Finland with size and growth data from birth through adulthood found that both birth ponderal index and BMI at 6 months were strongly associated with the incidence of obesity as an adult; and attributed this to accelerated weight gain through childhood.[21] Stettler et al. found that a change in CDC weight-for-age Z-score from birth – 4 months greater than 1 SD was strongly associated with obesity at 20 years old in African Americans.[22]

A few of these studies have been published since the original systematic reviews on the topic, but their underlying message does not differ. Our study also adds to the literature base on this since we are the first to use the WHO growth standards rather than a growth reference on this topic with adult overweight/obesity. Our ability to examine target ages and growth ranges throughout infancy provides a different and thorough approach to the topic. Expanded investigation of the range of birth through infancy allows this study to be compared to the earlier published studies which focused on certain points or ranges, while also providing distinctive data. As well, our data are unique in since they account for maternal and paternal BMI. The previous aforementioned reviews and studies largely included only maternal BMI as available. Our results demonstrate the importance of considering both of these aspects related to infant size and growth. Further research applying the WHO infant growth standards will provide insight into different status measures or ranges for rapid growth that best predict excess weight and other outcomes in adults.

The limitations of our study also need to be considered. In line with the general base of research on the topic there are numerous unmeasured environmental, lifestyle, behavioral, and genetic factors that may confound the association between infant size and growth and excess weight in young adulthood. A study with these aforementioned confounders would greatly enhance the understanding on this topic. Another limitation was the low prevalence of obesity which did not allow for any stratified analyses and provided somewhat unstable estimates. This low prevalence may be at least partially attributable to the historical nature of the cohort and many participants being born in and exposed to a different era environmentally. However, our stratified analyses by year of birth were consistent for overweight as an outcome. Furthermore, as in most birth cohorts and life course studies, there is loss to follow up and potential for sampling bias. We did not find any significant differences between baseline characteristics in participants who had and who did not have full parental data and were thus excluded; or between birth characteristics of those in this sample and the whole cohort. The results may also only be applicable to infants born appropriate for gestational age. Lastly, consideration that our study population is homogenous in both geographic and racial composition needs to be considered.

In conclusion, the data from this study demonstrate the importance of accounting for length in infant size and growth in relation to risk of later excess weight, and highlight how the whole range of infant growth may inform on risk of excess weight in adulthood. Overall, the results suggest that both static measures of size at target ages, and rapid growth intervals in infancy are associated with excess weight in young adulthood independent of birth-size and parental BMI. The strongest associations are in measures of rapid growth from birth to 24 months, 1 to 12, 18 and 24 months, and 3 to 9 and 12 months during the infancy period. Additionally, this study supports the clinical applicability of the WHO infant growth standards. Research incorporating the WHO infant growth standards with later obesity and other clinical risk markers will further inform understanding of these standards with health factors.

## Supporting Information

File S1 (DOC)
