# Supplementary material for: Systematic Examination of Infant Size and Growth Metrics as Risk Factors for Overweight in Young Adulthood
Source: PLoS One. 2013 Jun 20;8(6):e66994. doi: 10.1371/journal.pone.0066994 (PMC3688577; doi:10.1371/journal.pone.0066994)
Supplement: File S1 — (DOC) [file pone.0066994.s001.doc]

**Table S1: Odds ratio and 95% confidence interval of being obese (BMI ≥ 30 kg/m^2^)**

**as a young-adult (age 20-29 years) according to percentile of the WHO infant growth standards:**

**Infants ≥ 85^th^ percentile vs. < 85^th^ percentile**

| **Metric: WHO Weight for Age** | | | | | |
| --- | --- | --- | --- | --- | --- |
| **Outcome** | **Target Age (Months)** | **^b^N^obes^/ N^85th^** | **^1^OR (95% CI)** | **^2^OR (95% CI)** | **^3^OR (95% CI)** |
| **Obesity** | **0** | 8 / 47 | 6.97 (2.44-19.95 | NA | 4.93 (1.58-15.36) |
|  | **1** | 8 / 27 | 13.80 (4.50-42.34) | 9.16 (2.16-38.75) | 7.19 (1.59-32.52) |
|  | **3** | 1 / 9 | NA | NA | NA |
|  | **6** | 4 / 34 | 2.54 (0.78-8.34) | 1.42 (0.40-5.06) | 1.54 (0.39-6.05) |
|  | **9** | 9 / 72 | 3.81 (1.50-9.66) | 2.15 (0.74-6.21) | 1.65 (0.52-5.30) |
|  | **12** | 11 / 98 | 3.76 (1.52-9.29) | 2.39 (0.90-6.39) | 2.01 (0.68-5.90) |
|  | **18** | 10 / 100 | 3.43 (1.37-8.57) | 2.45 (0.94-6.41) | 2.23 (0.79-6.31) |
|  | **24** | 9 / 70 | 5.07 (1.95-13.20) | 3.46 (1.25-9.58) | 2.98 (1.00-8.92) |
| **Metric: WHO Weight for length** | | | | | |
|  | **0** | 1 / 16 | NA | NA | NA |
|  | **1** | 1 / 21 | NA | NA | NA |
|  | **3** | 0 / 15 | NA | NA | NA |
|  | **6** | 2 / 30 | NA | NA | NA |
|  | **9** | 5 / 51 | 2.79 (0.94-8.28) | 2.79 (0.92-8.41) | 2.79 (0.85-9.14) |
|  | **12** | 9 / 85 | 3.73 (1.49-9.50) | 4.17 (1.57-11.08) | 3.73 (1.32-10.56) |
|  | **18** | 12 / 102 | 6.57 (2.53-17.07) | 6.63 (2.44-18.01) | 6.29 (2.20-17.99) |
|  | **24** | 8 / 56 | 10.43 (3.41-31.92) | 7.98 (2.55-25.01) | 6.35 (1.65-21.82) |

-Overall N = 422, 22 obese young adults (BMI ≥ 30 kg/m^2^)

- OR (95% CI) represents estimate for ≥ 85^th^ percentile by target age compared to < 85^th^ percentile by target age (OR = 1.00)

- ^b^N^obes^/ N^85th^= Number who became obese as young adults in the group who were ≥ 85^th^ % at the target age

- Model 1: *^1^OR (95% CI)* = Adjusted for sex, gestational age at birth, age at adulthood obesity assessment, birth year,

- Model 2: *^2^OR (95% CI) =* Model 1 further adjusted for birth WHO weight-for-age or weight-for-length Z-score

- Model 3: ^3^*OR (95% CI)* = Model 2 plus maternal and paternal age and BMI

| **Table S2: Odds ratio and 95% confidence interval of being obese (BMI ≥ 30 kg/m^2^) as a young-adult (age 20-29 years) according to infant growth status between target ages: Rapid Weight-for-Length growth (∆ ≥ 0.67 Z score) vs. Non-rapid Weight-for-Length growth (∆ < 0.67 Z score)** | | | |
| --- | --- | --- | --- |
| **Rapid Growth - Metric: WHO Weight for length** | | | |
| **Outcome** | **Target Age (Months)** | **^a^N^obes^/ N^rapid^** | ***Rapid Growth***  **OR (95% CI)** |
| **Obese** | 0-1 | 3 / 119 | 0.78 (0.16-3.55) |
|  | 0-3 | 7 / 166 | 1.47 (0.42-5.13) |
|  | 0-6 | 11 / 222 | 2.43 (0.68-8.65) |
|  | 0-9 | 12 / 247 | 2.48 (0.68-9.04) |
|  | 0-12 | 14 / 272 | 3.88 (1.02-14.71) |
|  | 0-18 | 15 / 287 | 4.37 (1.14-16.69) |
|  | 0 -24 | 15 / 239 | 9.14 (2.34-37.73) |
|  |  |  |  |
|  | 1-3 | 1 / 37 | NA |
|  | 1-6 | 7 / 111 | 4.87 (1.33-17.88) |
|  | 1-9 | 13 / 174 | 5.69 (1.59-20.36) |
|  | 1-12 | 14 / 202 | 5.72 (1.63-20.03) |
|  | 1-18 | 15 / 224 | 6.39 (1.86-22.00) |
|  | 1-24 | 12 / 163 | 4.73 (1.46-15.33) |
|  |  |  |  |
|  | 3 - 6 | 3 / 39 | NA |
|  | 3 - 9 | 10 / 132 | 7.03 (2.00-24.72) |
|  | 3 - 12 | 12 / 195 | 3.76 (1.24-11.39) |
|  | 3 - 18 | 16 / 213 | 4.03 (1.35-12.03) |
|  | 3 -24 | 11 / 139 | 2.52 (0.88-7.21) |
|  |  |  |  |
|  | 6 – 9 | 1 / 6 | NA |
|  | 6 – 12 | 4 / 78 | 1.39 (0.34-5.71) |
|  | 6 – 18 | 10 / 137 | 1.49 (0.54-4.18) |
|  | 6 - 24 | 5 / 80 | 0.60 (0.16-2.21) |
|  |  |  |  |
|  | 12 - 24 | 2 / 18 | NA |

-Overall N = 422, 22 obese young adults (BMI ≥ 30kg/m^2^)

-Target age represents the beginning and end point of the growth period, e.g. 0-1 is birth to1 mo

-Rapid infant growth: (∆ ≥ 0.67 Z score of WHO weight-for-length standard) ~ change in centile on growth chart

- OR (95% CI) represents estimate for rapid growth (∆ ≥ 0.67) between target ages compared to non-rapid growth (< 0.67 Z score) (OR = 1.00)

- ^a^N^obes^/ N^rapid^ = Number who became obese as young adults in the group who experienced rapid growth between noted target age points

-All models adjusted for sex, gestational age at birth, age at adulthood obesity assessment, birth year, maternal and paternal age and BMI and birth weight-for-length Z-score

| **Table S3: Odds ratio and 95% confidence interval of being obese (BMI ≥ 30 kg/m^2^) as a young-adult (age 20-29 years) according to infant growth status between target ages: Rapid Weight-for-Age growth (∆ ≥ 0.67 Z score) vs. Non-rapid Weight-for-Age growth (∆ < 0.67 Z score)** | | | |
| --- | --- | --- | --- |
| **Rapid Growth - Metric: WHO Weight for Age** | | | |
| **Outcome** | **Target Age (Months)** | **^a^N^obes^/ N^rapid^** | ***Rapid Growth***  **OR (95% CI)** |
| **Obese** | 0-1 | 0 / 2 | NA |
|  | 0-3 | 0 / 13 | NA |
|  | 0-6 | 4 / 73 | 3.29 (0.81-13.33) |
|  | 0-9 | 5 / 134 | 1.00 (0.28-3.51) |
|  | 0-12 | 9 / 170 | 2.46 (0.79-7.66) |
|  | 0-18 | 8 / 168 | 1.91 (0.62-5.90) |
|  | 0 -24 | 7 / 138 | 2.91 (0.83-10.14) |
|  |  |  |  |
|  | 1-3 | 0 / 6 | NA |
|  | 1-6 | 3 / 71 | NA |
|  | 1-9 | 6 / 165 | 1.13 (0.34-3.75) |
|  | 1-12 | 10 / 211 | 1.58 (0.52-4.76) |
|  | 1-18 | 10 / 208 | 1.28 (0.44-3.76) |
|  | 1-24 | 8 / 163 | 1.61 (0.51-5.07) |
|  |  |  |  |
|  | 3 - 6 | 3 / 76 | NA |
|  | 3 - 9 | 12 / 260 | 1.23 (0.42-3.57) |
|  | 3 - 12 | 13 / 305 | 0.80 (0.28-2.28) |
|  | 3 - 18 | 16 / 282 | 1.86 (0.62-5.56) |
|  | 3 -24 | 12 / 222 | 1.61 (0.57-4.55) |
|  |  |  |  |
|  | 6 – 9 | 3 / 34 | NA |
|  | 6 – 12 | 10 / 151 | 2.70 (0.94-7.75) |
|  | 6 – 18 | 9 / 151 | 2.16 (0.77-6.06) |
|  | 6 - 24 | 7 / 117 | 1.72 (0.59-4.99) |
|  |  |  |  |
|  | 12 - 24 | 1 / 9 | NA |

-Overall N = 422, 22 obese young adults (BMI ≥ 30kg/m^2^)

-Target age represents the beginning and end point of the growth period, e.g. 0-1 is birth to1 mo

-Rapid infant growth: (∆ ≥ 0.67 Z score of WHO weight-for-length standard) ~ change in centile on growth chart

- OR (95% CI) represents estimate for rapid growth (∆ ≥ 0.67) between target ages compared to non-rapid growth (< 0.67 Z score) (OR = 1.00)

- ^a^N^obes^/ N^rapid^ = Number who became obese as young adults in the group who experienced rapid growth between noted target age points

-All models adjusted for sex, gestational age at birth, age at adulthood obesity assessment, birth year, maternal and paternal age and BMI and birth weight-for-age Z-score
